# Serum uric acid to high-density lipoprotein cholesterol ratio predicts all-cause mortality in adults with metabolic dysfunction associated steatotic liver disease

**DOI:** 10.1038/s41598-025-94651-5

**Published:** 2025-04-02

**Authors:** Yingyong Ou, Zihan Qin, Pinze Wang, Fan Zou

**Affiliations:** 1 Department of Respiratory and Critical Care Medicine, Affiliated Hospital of Zunyi Medical University, Zunyi, China; 2 Hebei Medical University, Shijiazhuang, 050017 China

**Keywords:** Metabolic dysfunction-associated steatotic liver disease, Serum uric acid, High-density lipoprotein cholesterol, Serum uric acid-to-high-density lipoprotein cholesterol ratio, All-cause mortality, NHANES, Outcomes research, Medical research, Risk factors

## Abstract

Metabolic dysfunction-associated steatotic liver disease (MASLD) is one of the most prevalent chronic metabolic diseases worldwide. While serum uric acid (SUA) and high-density lipoprotein cholesterol (HDL) are individually associated with the development of MASLD, the prognostic effect of the UA, HDL and SUA-to-HDL ratio (UHR) on the all-cause mortality of MASLD patients remains unexplored. This study utilized data from 4280 MASLD patients in the National Health and Nutrition Examination Survey (NHANES) from 1999 to 2018. UHR was calculated by dividing SUA by HDL, and its association with all-cause mortality was assessed using Cox proportional hazards models. Adjustments were made for demographic, lifestyle, and clinical factors. A one-standard-deviation increase in UA or UHR was associated with a 19% (HR 1.19, 95% CI 1.08–1.31, *P* < 0.001) or 18% (HR 1.18; 95% CI 1.07–1.30; *P* < 0.001) higher risk of all-cause mortality of MASLD participants respectively, while no association was found between HDL and mortality. SUA and UHR are promising predictors of all-cause mortality in MASLD patients, offering clinicians a valuable biomarker for related risk stratification. Its inclusion in clinical assessments could guide interventions and improve prognosis, advancing management for MASLD patients.

## Introduction

Nonalcoholic fatty liver disease (NAFLD) is one of the most prevalent chronic diseases worldwide, affecting approximately one-quarter of the global population, with its prevalence, incidence, and mortality rates continuing to rise^1^. It is progressively recognized as a major contributor to liver-related conditions such as decompensated cirrhosis, fibrosis, and hepatocellular carcinoma (HCC)^2^. Additionally, it is established as a high-risk factor for chronic extrahepatic complications, including type 2 diabetes mellitus (DM), cardiovascular disease (CVD), chronic kidney disease (CKD), metabolic syndrome, and premature mortality^3,4^. This condition significantly impacts the quality of life and imposes a substantial economic burden on populations, including those in the United States. Recently, following the Delphi consensus process, the term fatty liver disease (FLD) has been replaced by steatotic liver disease (SLD), with metabolic dysfunction-associated steatotic liver disease (MASLD) emerging as a new designation to mitigate potential stigma associated with NAFLD and enhance patient awareness^5^. With a comprehensive understanding of the various etiologies of steatosis and a detailed emphasis on metabolic dysregulation, diagnosing MASLD enhances our ability to evaluate its epidemiological significance, identify relevant biomarkers, advance drug development, and inform health policy initiatives^5^.

Previous studies have indicated that various chronic metabolic disorders, including dyslipidemia, CKD, and diabetes, are strongly associated with an increased mortality risk in patients with MASLD^6,7^. As is well known, high-density lipoprotein cholesterol (HDL), a conducive plasma lipoprotein, has been considered to have important antioxidant and anti-inflammatory functions^8^. Meanwhile, serum uric acid (SUA), the end product of purine metabolism in the liver, has also been associated with metabolic syndrome^9^. Most recently, the serum uric acid-to-HDL ratio (UHR) has been identified as a novel biomarker reflecting levels of inflammation and metabolic dysregulation^10–12^. It has also been shown to be a better predictor of NAFLD occurrence compared to the use of SUS or HDL-C alone^13^. To date, while some studies have explored the impact of HDL or SUA alone on the mortality prognosis of MASLD patients^14–16^, the conclusions remain inconsistent, and no studies have yet examined the prognostic significance of UHR in the mortality of MASLD patients.

Therefore, our research aims to fill this gap by exploring the prognostic impact and significance of elevated UHR on all-cause mortality in MASLD patients, based on the data from the National Health and Nutrition Examination Survey (NHANES). This is a preliminary retrospective study that may offer additional insights into the clinical progression of MASLD and its influence on patient mortality.

## Methods

### Data source and study population

The NHANES is an ongoing series of intricate and multistage surveys designed to provide a nationally representative assessment of the health and nutritional status of the noninstitutionalized civilian population in the United States. Comprehensive details regarding the survey methodologies and analytic guidelines can be found in NHANES documentation data collection involved conducting home interviews to gather extensive information on various health-related topics, including demographic, socioeconomic, and general health factors. This was followed by blood sample collection at a mobile examination center. The NHANES protocol was reviewed and approved by the Research Ethics Review Board of the National Center for Health Statistics (NCHS), and informed consent was obtained from all participants.

**Fig. 1:**
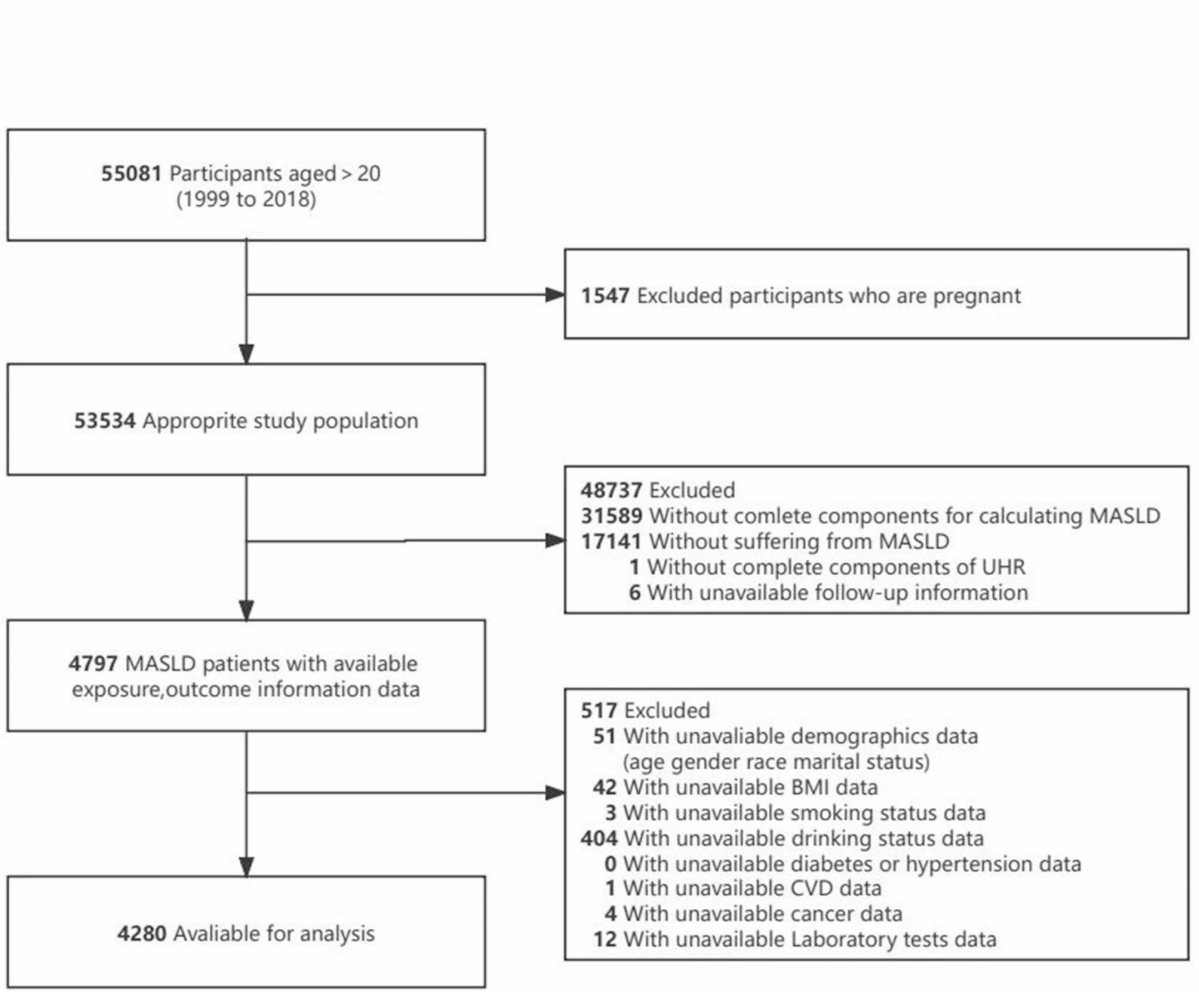
Flow diagram of the sample selection from the National Health and Nutrition Examination Survey (NHANES) 2019–2018. *MASLD* metabolic dysfunction-associated steatotic liver disease, *BMI* body mass index, *CVD* cardiovascular disease.

This cohort study utilized data from the National Health and Nutrition Examination Survey (NHANES) conducted between 1999 and 2018, focusing on adults aged 20 years or older. Initially, 55,081 participants were included, with 1547 excluded due to pregnancy. The remaining 53,534 individuals were assessed for MASLD, in which 31,589 participants without complete MASLD data and 17,141 without MASLD diagnosis were further excluded. Then, exclusions were made for participants with missing UHR data and follow-up data, and 4797 MASLD patients remained. After excluding other 517 individuals with incomplete demographic, clinical, or laboratory data, the final cohort included 4280 MASLD patients (Fig. 1).

### Assessment of MASLD

In most interview cycles, direct ultrasonographic assessments for hepatic steatosis were not conducted. As a result, hepatic steatosis was estimated using the Fatty Liver Index (FLI), a validated tool recognized for its high sensitivity and specificity in assessing SLD^17,18^. The FLI is calculated as previous study^19^. Based on previous research, participants with an FLI ≥ 30 were classified as having SLD^5^. To satisfy the diagnostic criteria for MASLD as outlined by the Delphi consensus, individuals with viral hepatitis, drug- or medication-induced liver disease, genetic liver disorders, autoimmune liver disease, alcohol-related liver disease, or alcohol consumption of ≥ 30 g/day for men and ≥ 20 g/day for women were excluded from the study^20^. MASLD was defined as SLD accompanied by at least one cardiometabolic risk factor: (1) BMI ≥ 25 kg/m^2^ or WC ≥ 94 cm for males and ≥ 80 cm for females; (2) Fasting blood glucose (FBG) ≥ 100 mg/dL, 2-h post-load glucose levels ≥ 140 mg/dL, hemoglobin A1c ≥ 5.7%, or a diagnosis of diabetes mellitus (DM) or current treatment with hypoglycemic agents; (3) Blood pressure ≥ 130/85 mmHg or current antihypertensive treatment; (4) Fasting plasma triglycerides ≥ 150 mg/dL or current lipid-lowering therapy; (5) Plasma HDL cholesterol < 40 mg/dL for males and < 50 mg/dL for females, or current lipid-lowering therapy^21^.

### Definitions of UHR

Blood samples were collected from participants in a fasting state during the morning hours to measure both HDL cholesterol and SUA levels. The procedure for HDL cholesterol quantification involved the addition of a magnesium sulfate/dextran solution to the sample, which resulted in the formation of water-soluble complexes with non-HDL cholesterol, thereby preventing their interaction with the reagents in the subsequent steps. Following this, HDL cholesterol esters were enzymatically hydrolyzed to HDL cholesterol through the action of polyethylene glycolesterase. The generated hydrogen peroxide then underwent a reaction with 4-aminoantipyrine and HSDA, producing a chromogenic compound that could be detected at a wavelength of 600 nm using photometric analysis. For the determination of SUA concentrations, the timed endpoint method was employed using a DxC800 automated chemistry analyzer. SUA was enzymatically oxidized by uricase to yield allantoin and hydrogen peroxide. The resultant hydrogen peroxide then reacted with 4-aminoantipyrine (4-AAP) and 3,5-dichloro-2-hydroxybenzenesulfonate (DCHBS) under the catalysis of peroxidase, leading to the formation of a colored product. This product was subsequently quantified photometrically at 520 nm to determine SUA concentrations. UHR was calculated by dividing the uric acid concentration (mg/dL) by the HDL cholesterol concentration (mg/dL) and multiplying the result by 100^22,23^.

### Assessment of outcomes

The primary endpoint of this study was all-cause mortality. Participants from the NHANES 2019–2018 cohort were prospectively monitored from their enrollment date up until December 31, 2019^24^. Mortality data were obtained by the NCHS through matching with death certificate records in the National Death Index. This matching process utilized key identifiers, including social security number, full name, date of birth, race/ethnicity, gender, birth status, and residential information^25^. All-cause mortality data were analyzed in accordance with the International Classification of Diseases, 10th Revision (ICD-10).

### Covariates

Demographic data of participants with MASLD such as age, gender, race and marital status were extracted from the NHANES database. BMI was calculated as weight (kg) divided by height squared (m^2^), then it was categorized into three groups: under 25 kg/m^2^, 25–30 kg/m^2^, and 30 kg/m^2^ or higher. Additionally, lifestyle factors and comorbidity history were documented, including smoking status, alcohol consumption, and medical history of diabetes mellitus (DM), hypertension, cancer, and cardiovascular disease (CVD). Smoking status was classified into current, former, and never smokers, with “never smokers” having smoked fewer than 100 cigarettes, “former smokers” having smoked more than 100 cigarettes previously, and “current smokers” actively smoking at the time of the interview. Drinkers (Yes) and non-drinkers (No) were defined according to whether they consumed 12 drinks or more per year. Comorbidities were ascertained by 7 separate questions of the form: “Have you ever been told by a doctor that you have [diabetes/hypertension/congestive heart failure/coronary heart disease/angina or angina pectoris/heart attack/cancer or malignancy]?” These variables were used as dichotomous variables according to the responses of “yes” or “no”. Laboratory assessments were conducted, capturing levels of alanine aminotransferase (ALT), aspartate aminotransferase (AST), alkaline phosphatase (ALP), gamma-glutamyl transferase (GGT), total cholesterol (TC), triglycerides (TG), total bilirubin (TB), and serum creatinine (SCR).

### Statistical analyses

For the analysis, we adhered to the NHANES database analytic guidelines (https://wwwn.cdc.gov/nchs/nhanes/tutorials/weighting.aspx, accessed on March 4, 2024), which recommend the use of sample weights to ensure that estimates are representative of the U.S. civilian non-institutionalized population. All statistical analyses accounted for sampling weights, clustering, and stratification to properly estimate variances and ensure the representativeness of the U.S. population with MASLD. The normality of continuous variables was assessed using the Kolmogorov-Smirnov test. Continuous variables were reported as either the mean (standard deviation, SD) or median (interquartile range, IQR), depending on their distribution, and categorical variables were expressed as counts (weighted percentages). For comparisons of continuous variables, the Student’s *t*-test or Kruskal–Wallis test was used, while categorical variables were compared using the Chi-square test.

The associations between HDL, UA, and UHR with all-cause mortality among MASLD participants were assessed using Cox proportional hazards models. Statistical significance was assessed by evaluating the adjusted hazard ratios (HR) in comparison to 1.0, along with their corresponding 95% confidence intervals (CI). Covariate adjustments were made based on prior research on MASLD survival^26–28^. Model 1 was unadjusted, while Model 2 included adjustments for age and gender. The fully adjusted model accounted for multiple factors, including age, gender, race, marital status, BMI, smoking habits, alcohol consumption, diabetes, hypertension, history of CVD, cancer history, as well as levels of albumin, ALT, AST, ALP, GGT, TC, TG, TB, and SCR.

To assess the dose-response relationship between UHR and all-cause mortality in individuals with MASLD, restricted cubic spline (RCS) models were utilized. The selection of knots for the RCS curves was based on the minimization of the Akaike Information Criterion (AIC). Subgroup and interaction analyses stratified by age, gender, BMI and common comorbidities were also performed to assess the robustness of the association between SUA, UHR and all-cause mortality of adults with MASLD.

All analyses were conducted using R (http://www.R-project.org, The R Foundation) and Free Statistics software versions 1.9. A two-tailed p-value < 0.05 was considered statistically significant.

## Result

### Baseline characteristics

Between 1999 and 2018, a total of 4280 MASLD patients were included, with a mean age of 57.5 years (SD 15.88). In terms of gender distribution, male participants (55.14%) slightly outnumbered female participants (44.86%). Regarding racial distribution, the majority of MASLD patients were non-Hispanic white (48.53%) while non-Hispanic black patients accounted for 11.78%. Concerning lifestyle habits, 32.73% of the patients were former smokers. 45.89% of the patients reported current alcohol consumption. In terms of comorbidities, 39.67% of the patients had diabetes mellitus, 61.92% had a history of hypertension, 18.41% had CVD and 12.85% had cancer. The mean UHR was 14.06 (SD 5.44), the mean SUA level was 6.02 (SD 1.46), and the mean HDL cholesterol level was 46.06 (SD 12.03). During the follow-up period, deceased patients (908 cases) exhibited significantly different characteristics compared to survivors (3372 cases). Deceased patients had a significantly higher mean age (69.99 years vs. 54.13 years) and a higher proportion of males (59.80% vs. 53.88%). Regarding racial distribution, a higher proportion of deceased patients were non-Hispanic white (62.33% vs. 44.81%,). Additionally, deceased patients had significantly higher proportions of former smokers (45.93% vs. 29.18%), hypertensive patients (76.43% vs. 58.01%), and those with cardiovascular disease (35.68% vs. 13.76%) (All *P* < 0.05), indicating a tendency towards adverse habits and comorbidities (Table 1).

**Table 1:** Baseline characteristics of adults with MASLD.

Variables	Total (*n* = 4280)	Survivors (*n* = 3372)	Non-survivors (*n* = 908)	*P*
Age (year, mean (SD))	57.50 (15.884)	54.13 (15.278)	69.99 (11.156)	< 0.001
Gender, n (%)				0.001
Female	1920 (44.86)	1555 (46.12)	365 (40.20)	
Male	2360 (55.14)	1817 (53.88)	543 (59.80)	
Race, n (%)				< 0.001
Mexican American	1013 (23.67)	842 (24.97)	171 (18.83)	
Non-Hispanic Black	504 (11.78)	411 (12.19)	93 (10.24)	
Non-Hispanic White	2077 (48.53)	1511 (44.81)	566 (62.33)	
Other Hispanic	369 (8.62)	321 (9.52)	48 (5.29)	
Other Race	317 (7.41)	287 (8.51)	30 (3.30)	
Marry, n (%)				< 0.001
Living with partner	213 (4.98)	191 (5.66)	22 (2.42)	
Married	2633 (61.52)	2122 (62.93)	511 (56.28)	
Never married	399 (9.32)	352 (10.44)	47 (5.18)	
Widowed, divorced, or separated individuals	1035 (24.18)	707 (20.97)	328 (36.12)	
BMI, kg/m^2^, n (%)				< 0.001
< 25	239 (5.58)	143 (4.24)	96 (10.57)	
25–30	1207 (28.20)	906 (26.87)	301 (33.15)	
≥ 30	2834 (66.21)	2323 (68.89)	511 (56.28)	
Smoke, n (%)				< 0.001
Former	1401 (32.73)	984 (29.18)	417 (45.93)	
Never	2346 (54.81)	1973 (58.51)	373 (41.08)	
Now	533 (12.45)	415 (12.31)	118 (13)	
Drink, n (%)				< 0.001
Former	1442 (33.69)	1055 (31.29)	387 (42.62)	
Never	874 (20.42)	684 (20.28)	190 (20.93)	
Now	1964 (45.89)	1633 (48.43)	331 (36.45)	
DM, n (%)				< 0.001
No	2582 (60.33)	2149 (63.73)	433 (47.69)	
Yes	1698 (39.67)	1223 (36.27)	475 (52.31)	
Hypertension, n (%)				< 0.001
No	1630 (38.08)	1416 (41.99)	214 (23.57)	
Yes	2650 (61.92)	1956 (58.01)	694 (76.43)	
CVD, n (%)				< 0.001
No	3492 (81.59)	2908 (86.24)	584 (64.32)	
Yes	788 (18.41)	464 (13.76)	324 (35.68)	
Cancer, n (%)				< 0.001
No	3730 (87.15)	3010 (89.26)	720 (79.30)	
Yes	550 (12.85)	362 (10.74)	188 (20.70)	
Albumin (g/L, mean (SD))	41.69 (3.15)	41.75 (3.14)	41.44 (3.20)	0.008
ALT (U/L, median [IQR])	25.00 [19.00, 33.00]	25.00 [20.00, 35.00]	21.00 [17.00, 28.00]	< 0.001
AST (U/L, median [IQR])	24.00 [20.00, 28.00]	24.00 [20.00, 29.00]	24.00 [19.00, 28.00]	0.219
ALP (U/L, median [IQR])	74.00 [61.00, 90.00]	73.00 [60.00, 89.00]	77.00 [64.00, 93.00]	< 0.001
GGT (U/L, median [IQR])	28.00 [20.00, 42.00]	28.00 [20.00, 41.00]	28.00 [19.00, 43.00]	0.636
TC (mg/dL, median [IQR])	192.00 [166.00, 221.00]	193.00 [167.00, 221.00]	189.00 [162.00, 220.00]	0.023
TG (mg/dL, median [IQR])	146.00 [105.00, 206.00]	147.00 [106.00, 206.00]	142.00 [102.00, 208.00]	0.270
TB (mg/dL, median [IQR])	0.60 [0.50, 0.80]	0.60 [0.50, 0.80]	0.70 [0.60, 0.90]	< 0.001
SCR (mg/dL, median [IQR])	0.88 [0.71, 1.02]	0.85 [0.70, 1.00]	0.97 [0.80, 1.19]	< 0.001
HDL (mg/dL, mean (SD))	46.06 (12.03)	45.79 (11.66)	47.05 (13.26)	0.010
SUA (mg/dL, mean (SD))	6.02 (1.46)	5.95 (1.39)	6.24 (1.66)	< 0.001
UHR (%, mean (SD))	14.06 (5.44)	13.96 (5.25)	14.43 (6.05)	0.033

*MASLD* metabolic dysfunction-associated steatotic liver disease, *BMI* body mass index, *DM* diabetes mellitus, *CVD* cardiovascular disease, *ALT* alanine aminotransferase, *AST* aspartate aminotransferase, *ALP* alkaline phosphatase, *GGT* gamma-glutamyl transpeptidase, *TC* total cholesterol, *TG* triglyceride, *TB* total bilirubin, *SCR* serum creatinine, *HDL* high-density lipoprotein, *SUA* serum uric acid, *UHR* uric acid and high-density lipoprotein cholesterol ratio.

### Association between UHR and all-cause mortality

According to Cox regression analysis, elevated UHR was significantly associated with all-cause mortality in MASLD patients. In the unadjusted model (Model 1), no significant association was observed between a one-standard-deviation increase in UHR and all-cause mortality (HR 1.03, 95% CI 0.94–1.14, *P* = 0.526). However, after adjusting for age, gender, and other variables (Model 2), UHR was significantly associated with an increased risk of all-cause mortality (HR 1.26, 95% CI 1.14–1.40, *P* < 0.001), and this association remained significant in the fully adjusted model (Model 3: HR 1.18, 95% CI 1.07–1.30, *P* < 0.001). Similarly, UA showed a consistent positive association with an increased risk of all-cause mortality across all models, with the most significant results observed in Model 3 (HR 1.19, 95% CI 1.08–1.31, *P* < 0.001). In contrast, HDL was inversely associated with all-cause mortality after adjustment (Model 2: HR 0.91, 95% CI 0.83–0.99, *P* = 0.032), although this association was not significant in the fully adjusted model (Model 3: HR 0.98, 95% CI 0.89–1.07, *P* = 0.629).

Quartile analysis for UHR indicated that patients in the highest quartile (Q5) had a significantly increased risk of all-cause mortality (Model 3: HR 1.40, 95% CI 1.03–1.89, *P* = 0.030). Trend tests showed that higher UHR quartiles were significantly associated with increased mortality risk across all models. Similarly, patients with the highest UA quartile (Q5) had a significantly higher risk of all-cause death. Trend tests also showed a strong association between a higher UA quartile and an increased risk of death (Table 2).

**Table 2:** Association between UHR score and all-cause mortality of adults with MASLD.

	Model 1		Model 2		Model 3	
	HR (95% CI)	*P* value	HR (95% CI)	*P* value	HR (95% CI)	*P* value
HDL (per SD increase)	1.15 (1.06–1.25)	< 0.001	0.91 (0.83–0.99)	0.032	0.98 (0.89–1.07)	0.629
HDL quartiles						
Q1	1 [Reference]		1 [Reference]		1 [Reference]	
Q2	0.88 (0.66–1.16)	0.358	0.72 (0.54–0.97)	0.029	0.84 (0.63–1.10)	0.200
Q3	1.07 (0.82–1.40)	0.603	0.77 (0.59–1.00)	0.054	0.94 (0.73–1.20)	0.622
Q4	1.24 (0.93–1.64)	0.137	0.74 (0.54–1.00)	0.049	0.94 (0.69–1.29)	0.719
Q5	1.26 (0.95–1.67)	0.109	0.63 (0.46–0.87)	0.005	0.83 (0.61–1.14)	0.252
Trend test		0.007		0.014		0.590
SUA (per SD increase)	1.16 (1.05–1.28)	0.003	1.20 (1.08–1.32)	< 0.001	1.19 (1.08–1.31)	< 0.001
SUA quartiles						
Q1	1 [Reference]		1 [Reference]		1 [Reference]	
Q2	0.78 (0.59–1.03)	0.078	0.78 (0.60–1.02)	0.069	0.90 (0.67–1.21)	0.473
Q3	0.75 (0.57–0.98)	0.032	0.77 (0.59–1.00)	0.048	0.91 (0.70–1.18)	0.469
Q4	0.70 (0.54–0.92)	0.009	0.76 (0.59–0.97)	0.030	0.90 (0.68–1.19)	0.466
Q5	1.13 (0.89–1.44)	0.316	1.24 (0.96–1.58)	0.095	1.34 (1.00–1.79)	0.049
Trend test		0.310		0.060		0.032
UHR (per SD increase)	1.03 (0.94–1.14)	0.526	1.26 (1.14–1.40)	< 0.001	1.18 (1.07–1.30)	< 0.001
UHR quartiles						
Q1	1 [Reference]		1 [Reference]		1 [Reference]	
Q2	0.86 (0.63–1.17)	0.346	0.96 (0.72–1.28)	0.782	1.03 (0.78–1.38)	0.820
Q3	0.77 (0.58–1.02)	0.072	0.97 (0.75–1.25)	0.818	1.09 (0.84–1.42)	0.514
Q4	0.72 (0.53–0.99)	0.043	1.11 (0.83–1.49)	0.463	1.13 (0.83–1.53)	0.434
Q5	0.89 (0.67–1.17)	0.399	1.53 (1.12–2.09)	0.008	1.40 (1.03–1.89)	0.030
Trend test		0.305		0.003		0.026

*Model 1* Crude Model, *Model 2* Adjusted for age, gender, *Model 3* Adjusted for age, gender, race and ethnicity, marital status, BMI, smoking status, drinking status, diabetes, hypertension, CVD history, cancer history, albumin, ALT, AST, ALP, GGT, TC, TG, TB, and SCR, *MASLD* metabolic dysfunction-associated steatotic liver disease, *HR* hazard ratio, *CI* confidence interval, *SD* standard deviation, *Q* quartile, *HDL* high-density lipoprotein, *SUA* serum uric acid, *UHR* uric acid and high-density lipoprotein cholesterol ratio, *BMI* body mass index, *CVD* cardiovascular disease, *ALT* alanine aminotransferase, *AST* aspartate aminotransferase, *ALP* alkaline phosphatase, *GGT* gamma-glutamyl transpeptidase, *TC* total cholesterol, *TG* triglyceride, *TB* total bilirubin, *SCR* serum creatinine.

**Fig. 2:**
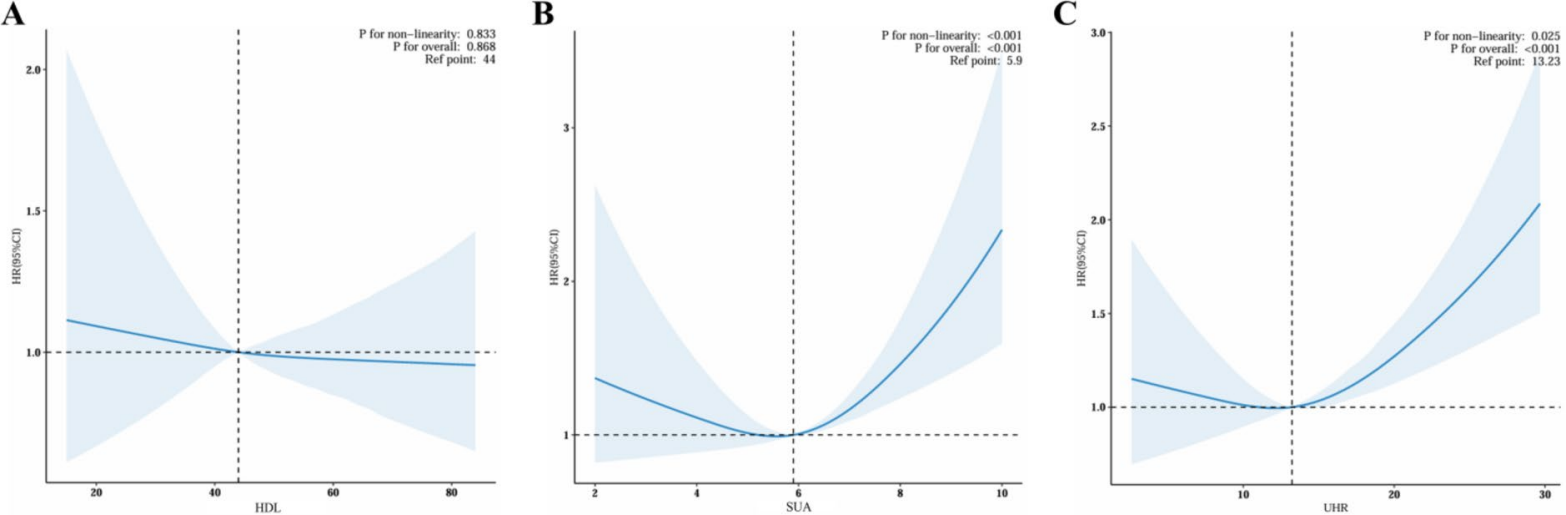
Restricted cubic splines reflect the dose-effect relationships between SUA, UHR with the all-cause mortality among adults with MASLD. (**A**) HDL; (**B**) SUA; (**C**) UHR. Adjusted for age, gender, race/ethnicity, marital status, body mass index, smoking status, alcohol consumption, diabetes, hypertension, CVD history, cancer history, albumin, ALT, AST, ALP, GGT, TC, TG, TB, and SCR. *MASLD* metabolic dysfunction-associated steatotic liver disease, *SUA* serum uric acid, *UHR* uric acid and high-density lipoprotein cholesterol ratio, *BMI* body mass index, *CVD* cardiovascular disease, *ALT* alanine aminotransferase, *AST* aspartate aminotransferase, *ALP* alkaline phosphatase, *GGT* gamma-glutamyl transpeptidase, *TC* total cholesterol, *TG* triglyceride, *TB* total bilirubin, *SCR* serum creatinine.

The adjusted multivariate restricted cubic spline (RCS) plot revealed no association was observed between HDL and all-cause mortality in MASLD adults (P for overall trend > 0.05, Fig. 2A). Nonlinear trends between SUA and all-cause mortality (P for nonlinearity < 0.001, P for overall trend < 0.001, Fig. 2B) and between UHR and all-cause mortality (P for nonlinearity = 0.025, P for overall trend < 0.001, Fig. 2C) among MASLD participants were also found, highlighting possible thresholds or saturation points beyond which mortality risk accelerates.

### Sensitivity analyses

**Fig. 3:**
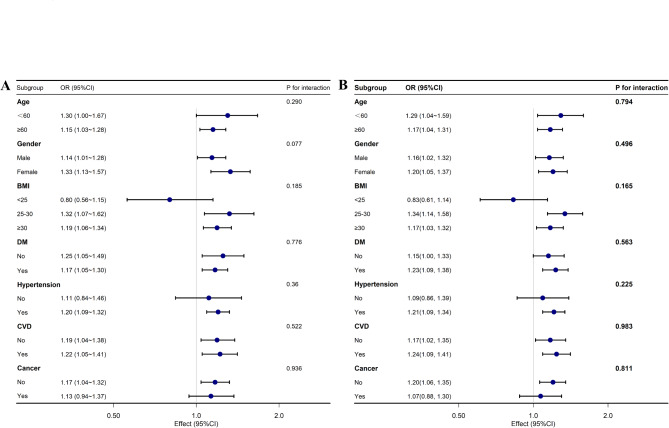
Association between SUA, UHR and All-Cause Mortality of adults with MASLD in different subgroups. (**A**) SUA; (**B**) UHR. Adjusted for age, gender, race/ethnicity, marital status, body mass index, smoking status, alcohol consumption, diabetes, hypertension, CVD history, cancer history, albumin, ALT, AST, ALP, GGT, TC, TG, TB, and SCR. *MASLD* metabolic dysfunction-associated steatotic liver disease, *SUA* serum uric acid, *UHR* uric acid and high-density lipoprotein cholesterol ratio, *BMI* body mass index, *CVD* cardiovascular disease, *ALT* alanine aminotransferase, *AST* aspartate aminotransferase, *ALP* alkaline phosphatase, *GGT* gamma-glutamyl transpeptidase, *TC* total cholesterol, *TG* triglyceride, *TB* total bilirubin, *SCR* serum creatinine.

The results of the subgroup analysis demonstrated no significant interaction between any of the subgroups (P for interaction > 0.05, Fig. 3). Specifically, the relationship was consistent across the stratified subgroups by age, sex, BMI, and the presence of comorbidities such as DM, hypertension, CVD, and cancer. These findings indicate that the association between UHR index and mortality is stable and does not vary meaningfully within different population groups.

## Discussion

Our cohort study indicates that, after accounting for potential confounders^29^, elevated levels of SUA and the UHR remain significantly positively correlated with all-cause mortality and adverse outcomes in patients with MASLD within American adult population. These findings underscore the clinical relevance of using SUA and UHR metrics for predicting treatment responses and assessing mortality risk in MASLD patients.

Recent evidence indicates that MASLD and NAFLD share similar clinical characteristics and mortality rates, supporting the potential utility of MASLD in future clinical practice^30^. Currently, we have identified two studies with limited evidence on SUA and NAFLD related mortality. One study involving the data from 1999 to 2014 NHANES database found that higher SUA levels were significantly associated with an increased risk of CVD mortality among NAFLD participants (per SD increment, HR 1.40, 95% CI 1.16–1.68), whereas no such association was observed in non-NAFLD participants^14^. A similar trend was observed for all-cause mortality. Our findings are consistent with these results; we observed that higher SUA levels were significantly associated with increased all-cause mortality risk in MASLD participants (per SD increment, HR 1.19, 95% CI 1.08–1.31, *P* < 0.001), and the fifth quintile of SUA (HR 1.34, 95% CI 1.00–1.79, *P* = 0.049) was independently associated with increased all-cause mortality in MASLD participants. However, Xinyi Yang et al. found in their study of the 1988–1994 NHANES database that after propensity score matching for age and sex, SUA was not associated with mortality in NAFLD patients (HR 1.00, 95% CI 1.00–1.00, *P* > 0.05), with similar results for cardiovascular and cancer-related deaths^31^. We speculate that the discrepancy may be due to the smaller sample size and the difference between the older database and the newer database. Additionally, prior research has suggested that lower HDL may be a risk factor for NAFLD^32,33^. However, our study did not find a significant association between HDL levels and mortality in MASLD patients.

Notably, Kosekli et al. were the first to introduce UHR as a strong predictor for metabolic syndrome, finding that UHR outperforms individual metrics of SUA or HDL in predicting the risk of NAFLD^34^. Additionally, literature indicates a high-risk association between UHR and the presence of atrial fibrillation in MASLD patients^35^. Although the positive correlation between UHR and MASLD has been established, the prognostic impact of the UHR index on this population remains unclear. This study addresses this gap by leveraging a representative national cohort. Specifically, after adjusting for a range of demographic characteristics, UHR (per SD increment, HR 1.18, 95% CI 1.07–1.30, *P* < 0.001) and the fifth quintile of UHR (per SD increment, adjusted HR 1.40, 95% CI 1.03–1.89, *P* = 0.030) are independently associated with increased all-cause mortality in MASLD participants, which was similar to the effect observed for SUA on all-cause mortality risk in MASLD.

We hypothesize that the underlying mechanisms contributing to these results may include the following. First, SUA plays a significant role in metabolic diseases, and hyperuricemia is closely associated with the development and progression of various metabolic disorders. Elevated SUA levels may affect mortality outcomes by promoting inflammation and oxidative stress. Specifically, SUA could contribute to endothelial dysfunction and atherosclerosis, thereby increasing the risk of cardiovascular events^36^. The study also indicated that SUA primarily promotes liver fat synthesis and accumulation through mitochondrial oxidative stress, and lowering SUA levels can inhibit the increase in cellular toxic substances and lipid peroxidation products in cells, thereby improving liver function and metabolic status in MASLD patients and potentially reducing the risk of death. Furthermore, elevated UHR has been significantly associated with insulin resistance^23^, which, in turn, has a substantial prognostic impact on all-cause mortality in MASLD patients^37^. Therefore, monitoring UHR levels may aid in identifying patients at higher risk for adverse outcomes, including mortality, and guide therapeutic interventions aimed at reducing hepatic and cardiovascular risks.

This study has several strengths. Firstly, to our knowledge, it is the first to investigate the prognostic impact of UHR on mortality outcomes in American adults with MASLD. Our findings highlight the clinical value of UA and UHR in the management of MASLD populations. Secondly, the study benefits from a substantial sample size and an adequate follow-up period to observe mortality outcomes, which enhances the statistical power and reliability of our results. Additionally, we adjusted for potential confounders to ascertain the independent association between UHR-related metrics and mortality outcomes in MASLD. Lastly, we conducted stratified analyses to elucidate the relationship between UHR and mortality across different subgroups within the study population, emphasizing the clinical significance of elevated UHR-related metrics.

However, several limitations should be addressed in future research. First, MASLD and liver fibrosis staging were not defined using the gold standard liver biopsy but rather by the Fatty Liver Index (FLI). Although FLI may offer advantages by primarily selecting MASLD patients^19^, it is not the optimal tool for distinguishing the specific causes of steatotic liver disease (SLD). Future studies which can validate our findings in cohorts with histologically confirmed MASLD participants need to be done. Second, we calculated UHR-related metrics using baseline data, which does not allow for an assessment of longitudinal changes in UHR-related metrics and MASLD clinical outcomes over time. Third, unmeasured confounders, including data on dietary patterns (e.g., fructose intake) and medications affecting UHR (e.g., allopurinol, statins) were unavailable. These factors may confound the observed associations which further studies should take them into consideration. Finally, since the study participants were exclusively from the United States, caution should be exercised when generalizing the results to diverse global populations. Future prospective longitudinal studies are needed to establish optimal cut-off values and provide stronger evidence to support the clinical application of UHR-related metrics in the management of adult MASLD.

## Conclusion

Our findings indicate that elevated levels of UA and UHR are associated with an increased risk of all-cause mortality in American adults with MASLD. UA and UHR have the potential to become simple and easily calculable clinical biomarkers for risk stratification in MASLD. Clinicians should consider monitoring UHR to guide early interventions targeting hyperuricemia or dyslipidemia. By identifying patients with elevated UA and UHR, healthcare providers may be able to implement more targeted and timely interventions, potentially improving survival outcomes for MASLD patients.

## Data Availability

Data availability statementPublicly available and de-identified data used in this analysis can be found in the CDC National Center for Health Statistics NHANES database at https://www.cdc.gov/nchs/nhanes.
